# Development of a Scoring Instrument for Identification of Pneumonitis in Older Lung Cancer Patients After Radiotherapy (POLCAR): A Protocol for a Prospective Trial

**DOI:** 10.3390/cancers17050807

**Published:** 2025-02-26

**Authors:** Dirk Rades, Inga Zwaan, Daphne Schepers-von Ohlen, Sabine Bohnet, Stefan Janssen, Julia Koeck, Justus Domschikowski, Charlotte Kristiansen, Marciana N. Duma, Silke Keerl, Tobias Bartscht, Nathan Y. Yu, Jon Cacicedo, Elisa M. Groh

**Affiliations:** 1 Department of Radiation Oncology, University Medical Center Schleswig-Holstein, Lübeck Campus, 23538 Lübeck, Germany; inga.zwaan@uksh.de (I.Z.); daphne.schepers-vonohlen@uksh.de (D.S.-v.O.); st-janssen@gmx.net (S.J.); elisamarie.groh@uksh.de (E.M.G.); 2 Radiation Oncology Department, University of the Basque Country, 48903 Barakaldo, Vizcaya (Basque Country), Spain; jon.cacicedofernandezbobadilla@osakidetza.eus; 3 Department of Pulmonology, University Medical Center Schleswig-Holstein, Lübeck Campus, 23538 Lübeck, Germany; sabine.bohnet@uksh.de; 4 Medical Practice for Radiotherapy and Radiation Oncology, 30161 Hannover, Germany; 5 Department of Radiotherapy, Malteser Hospital St. Franziskus, 24939 Flensburg, Germany; julia.koeck@malteser.org (J.K.); justus.domschikowski@malteser.org (J.D.); 6 Department of Oncology, Vejle Hospital, University Hospital of Southern Denmark, 7100 Vejle, Denmark; charlotte.kristiansen@rsyd.dk; 7 Department of Radiotherapy, Helios Hospital Schwerin, 19055 Schwerin, Germany; marciana.duma@helios-gesundheit.de; 8 Department of Hematology, Oncology and Stem Cell Transplantation, Helios Hospital Schwerin, 19055 Schwerin, Germany; silke.keerl@helios-gesundheit.de (S.K.); tobias.bartscht@helios-gesundheit.de (T.B.); 9 Department of Radiation Oncology, Mayo Clinic, Phoenix, AZ 85054, USA; yu.nathan@mayo.edu; 10 Radiation Oncology Department, Hospital Universitario Cruces, 48903 Barakaldo, Vizcaya (Basque Country), Spain; 11 Radiation Oncology Department, Biobizkaia Health Research Institute, 48903 Barakaldo, Vizcaya (Basque Country), Spain

**Keywords:** lung cancer, elderly patients, radiotherapy, chemoradiation, pneumonitis, scoring system

## Abstract

**Background/Objectives**: Pneumonitis caused by radiotherapy for lung cancer may be missed since it often occurs only several months later. In a previous trial including patients of any age, a scoring system was tested to facilitate the correct diagnosis of radiation pneumonitis. Since elderly lung cancer patients have a greater risk of developing this complication, a separate scoring system for this group appears useful. Our prospective multi-center trial (NCT06480734) investigates a specific tool for elderly patients irradiated for lung cancer. **Methods:** Patients aged ≥65 years with lung cancer will complete paper-based questionnaires and rate symptoms potentially caused by pneumonitis weekly during and up to 24 weeks following radiotherapy. The total score of this symptom-based scoring system ranging from 0 to 9 points is correlated to pneumonitis. The discriminative power of the scoring system is evaluated by calculating the area under the receiver operating characteristic curve. Optimality is defined as a cut-off score with sensitivity ≥90% and specificity ≥80%. Moreover, the Youden index will be applied. Fifty-nine patients are required for the full analysis set. Assuming 5% will not qualify for this set, 65 patients should be enrolled. Moreover, patient satisfaction with the scoring system is evaluated. If the dissatisfaction rate is >20%, the system needs modifications; if the dissatisfaction rate is >40%, it is considered not useful. An optimal cut-off score facilitating the diagnosis of pneumonitis and its discrimination from other lung diseases will contribute to a corresponding mobile application to be used by elderly lung cancer patients at home.

## Simple Summary

Radiation pneumonitis in lung cancer patients often occurs only several months after the treatment and, therefore, may be missed. Elderly lung cancer patients have a greater risk of developing radiation pneumonitis than younger patients. The present prospective multi-center trial (NCT06480734) evaluates a specific symptom-based scoring system for lung cancer in patients aged ≥65 years. It aims to identify the optimal cut-off score in order to facilitate the diagnosis of radiation pneumonitis and its discrimination from other lung diseases. In addition, patient satisfaction with the symptom-based scoring system will be assessed. The optimal cut-off score is a prerequisite for the development of a corresponding mobile application that can be used by elderly lung cancer patients at home.

## 1. Introduction

Lung cancer belongs to the most common types of solid cancer in Europe and Northern America [1]. Most patients with small cell lung cancer receive radiotherapy in combination with chemotherapy as definitive treatment [2]. Also, a considerable number of patients with advanced non-small cell lung cancer are treated with radiotherapy with or without concurrent chemotherapy [2]. Radiation pneumonitis is a possible subacute side effect of radiotherapy for lung cancer. Severe pneumonitis was reported to be fatal in approximately 2% of patients experiencing this adverse event [3].

Pneumonitis may occur up to 23 weeks following radiotherapy [4,5]. The symptoms may not be associated with the previously administered radiation treatment, and pneumonitis may be missed. Symptoms of pneumonitis generally include a cough, shortness of breath, and fever. In a previous prospective trial that included patients of any age, a scoring system was developed to contribute to a more precise and earlier detection of symptomatic radiation pneumonitis (grade ≥2) after radiotherapy for lung cancer [6]. In that trial, 0 to 3 scoring points were assigned to each of the three symptoms mentioned above. Thus, patient scores ranged between 0 and 9 points. The optimal cut-off score for the identification of pneumonitis was found to be 5 points. Moreover, an increase by 3 or more points from the baseline score was significantly associated with pneumonitis.

Since the previous trial was performed in lung cancer patients of any age, it is not clear whether its results also hold true for elderly patients aged 65 years or older [6]. This question needs to be answered to provide optimal diagnosis and treatment of pneumonitis for this growing age group. This idea is supported by the fact that several studies investigating pneumonitis after radiotherapy for lung cancer found that elderly patients had a higher risk of developing this complication [7,8,9,10,11,12,13]. In a retrospective study of our group performed in lung cancer patients aged ≥65 years, the prevalence of pneumonitis at 24 weeks following radiotherapy was 27.3% [14]. In those patients in whom the ipsilateral lung received a mean radiation dose of more than 13 Gy, the prevalence was 37.1%. For comparison, in another study, the prevalence of pneumonitis was 20.7% in patients aged ≤66 years [15].

The present prospective POLCAR trial investigates whether the results of the previous prospective trial can be generalized to elderly patients aged ≥65 years [6]. Its major goal is the identification of the optimal cut-off score to correctly diagnose pneumonitis and discriminate it from other lung diseases in elderly patients irradiated for lung cancer. In addition, the diagnostic value of an increase in the scoring points from baseline scores is evaluated. This study is a prerequisite for the development of a mobile application that can be used by patients at home to rate their symptoms possibly related to pneumonitis. This information will contribute to the decision whether pneumonitis is possible or likely and whether consultation of a pulmonologist or presentation to a hospital is required. This article reports on the study protocol of the POLCAR trial, including the objectives of the study, the eligibility criteria, planned assessments, interventions, information regarding sample size calculations, and the statistical methods used for primary and secondary outcomes.

## 2. Materials and Methods

This is a multi-center, single-arm prospective interventional study performed in two university hospitals, two academic teaching hospitals, and one private practice, which aims to establish the performance characteristics and develop a decision-algorithm of a symptom-based scoring system with respect to the identification of elderly patients developing pneumonitis after radiotherapy for lung cancer. The scoring system was created by experienced pulmonologists and successfully used in a previous trial [6].

### 2.1. Objectives and Endpoints

The primary objective is to assess the performance characteristics of the symptom-based scoring system for the detection of radiation pneumonitis in elderly patients with non-small cell lung cancer (NSCLC) or small cell lung cancer (SCLC). The receiver operating characteristic (ROC) curve is used to show the connection between sensitivity and specificity for every possible cut-off for the scoring system and select the optimal scoring point for the detection of radiation pneumonitis. The area under the ROC curve (AUC) is calculated to assess the diagnostic ability of the scoring system. Secondary objectives include positive and negative predictive values associated with each scoring point and patient satisfaction with the symptom-based scoring system (symptom-questionnaire, paper version). Based on the experiences of our previous trial and after discussion with the Ethics Committee, an assessment of quality of life was not considered necessary for the POLCAR trial [6]. It is not required for the primary objective and would mean additional stress for the patients.

### 2.2. Eligibility Criteria

The criteria for inclusion and exclusion are shown in Table 1.

### 2.3. Assessments

The following parameters will be assessed prior to radiotherapy: medical history, concomitant diseases (comorbidity), concomitant medication, physical examination, demographics, body height and weight, performance status, tumor stage and histology, upfront surgery, systemic anticancer treatment, and lung function. During the course of the study, symptoms of pneumonitis and adverse events other than pneumonitis will be assessed on an ongoing basis according to CTCAE v5.0 [16]. During the period of radiotherapy, patients are seen by medical staff members each day of radiation treatment. Following the course of radiotherapy, they are contacted weekly until the end of the study. So, it is unlikely that a patient is lost to follow-up. The timeline of the assessments is shown in Table 2.

### 2.4. Interventions

The patients receive the same radiotherapy or chemoradiation for lung cancer as they would have received without participation in the POLCAR trial. Radiotherapy is performed as volumetric-modulated arc therapy using conventional fractionation with doses per fraction of 2.0 Gy given five consecutive days per week and total doses ranging between 60 and 70 Gy [2]. The patients in this trial may receive any medical support required for adverse events, co-morbidities, or other conditions. Trial-specific interventions include completion of a paper-based questionnaire (symptom-based scoring system) once a week during and up to 24 weeks following radiotherapy and completion of a questionnaire rating the patient satisfaction with the symptom-based scoring system at the end of the radiotherapy course. The follow-up period of 24 weeks was chosen according to the results of previous studies showing that pneumonitis can occur up to 23 weeks following radiotherapy [4,5]. The English version of the questionnaire was properly translated into the languages of the participating centers.

The patients indicate and rate symptoms potentially caused by radiation pneumonitis, i.e., cough, dyspnea, and fever (Table 3), once a week by completing a paper-based questionnaire. During the radiotherapy course, the patients complete the questionnaire at the department of radiotherapy. After completion of radiotherapy, the information regarding the symptoms potentially related to pneumonitis will be obtained weekly by phone (to minimize hospital visits). The procedure of the telephone visits is clear, and uniformity is ensured. Support from caregivers is not allowed. Since the patients are contacted actively by staff members, the risk of loss to the follow-up is comparably low. In our previous trial, the rate of termination of follow-up due to the patient’s unavailability was 8.8% [6].

If the total score is higher than at baseline prior to the start of radiotherapy, the patients receive a follow-up call after 3 days or are asked to present at the corresponding hospital. If pneumonitis is suspected, the patients undergo lung function tests. The suspected diagnosis of pneumonitis is considered substantiated in case of a decrease in forced expiratory volume in 1 s (FEV1) and diffusing capacity of the lung for carbon monoxide (DLCO) to less than 75% from baseline values [16,17]. In this situation, the patients receive a chest X-Ray (which may be supplemented by a computed tomography). Radiation pneumonitis is considered confirmed if opacities confined to the irradiation fields are seen on chest X-Ray and/or ground-glass opacities (focal or nodular), consolidation, or both are seen on computed tomography [18,19]. The imaging findings are interpreted by experienced radiologists. If the diagnosis of pneumonitis grade ≥ 2 (CTCAE v5.0) is made, the patients generally receive treatment with prednisolone [5,16,20]. If the symptoms are caused by a medical condition other than pneumonitis, the patients are treated for this situation. Finally, the total score of the symptom-based scoring system is correlated to pneumonitis (yes or no). Since the scoring tool is easy to use, consistent assessment across multiple centers appears secured. This assumption is supported by the experiences from a previous prospective trial [6].

At the end of the radiotherapy course, the patients will complete a questionnaire regarding their satisfaction with the paper-based version of the scoring system, modified according to the User Experience Questionnaire, a widely used and reliable tool [21,22]. If the dissatisfaction rate is >20%, the scoring system needs to be modified, and if the dissatisfaction rate is >40%, it is considered not useful. If patients withdraw their consent to participate in the trial, study-specific interventions are stopped. In case of adverse events, which do not allow patients to complete subsequent questionnaires, study-specific interventions are postponed or discontinued, if necessary.

### 2.5. Sample Size Calculations

The main goal of this trial is to evaluate the usefulness of a new symptom-based scoring system with respect to the identification of elderly lung cancer patients developing pneumonitis after radiotherapy. The discriminative power of the symptom-based score will be assessed by calculating the AUC. The sample size is calculated to achieve acceptable precision in the estimation of the AUC measured by means of the 95% confidence interval by incorporating a pre-specified probability, which is referred to as assurance, of achieving the desired lower confidence limit [23].

The sample size calculations are based on a two-sided significance level of 5%, an AUC of 0.9, a pre-specified lower bound of the confidence interval of 0.7, an assurance probability of 80%, a ratio between negative and positive cases of 2.33 (70% of patients without and 30% with radiation pneumonitis). Based on these assumptions, 59 patients (18 with and 41 without radiation pneumonitis) are required within the Full Analysis Set. Assuming that 5% of patients will not qualify for the Full Analysis Set, a total of 62 patients should be enrolled. The drop-out rate includes patients who experience difficulties in completing questionnaires due to neurocognitive decline during the study period. The Full Analysis Set includes all the subjects who started radiotherapy.

A pneumonitis rate of 30% has been considered appropriate for the sample size calculation since, in a previous retrospective study investigating elderly patients irradiated for lung cancer, 27.3% of the patients developed pneumonitis [14]. Moreover, this rate is expected to be higher in the POLCAR trial due to the weekly assessment of pneumonitis for 24 weeks compared to the standard follow-up consisting of one visit about 6–8 weeks following radiotherapy only. All the lung cancer patients aged ≥ 65 years will be screened at each participating center. The recruitment of all 62 patients should be completed within 15 months. The treatment period will be 6–7 weeks, and the follow-up period 24 weeks. This equals a total running time for the trial of 22 months.

### 2.6. Statistical Methods for Primary and Secondary Outcomes

#### 2.6.1. Primary Objective

The primary objective is to assess the performance characteristics of the symptom-based scoring system for the detection of radiation pneumonitis. An evaluation will be performed in patients available for assessment of the primary endpoint who have completed at least 10 questionnaires regarding their symptoms. To allow for patient-based analyses, the multiple score values documented for each patient over time will be reduced to one clinically relevant, patient-specific value only. Thus, for patients without radiation pneumonitis during the study, the maximum score value documented throughout the study will be used, and for patients with pneumonitis, we use the value documented at the time of diagnosis. These patient-specific values represent the fundamental units for all further statistical analyses. First of all, sensitivity and specificity will be estimated for every possible cut-off value of the symptom-based scoring system.

The ROC curve is used to graphically show the connection between sensitivity and specificity. ROC is defined as the plot of sensitivity versus 1-specificity (false-positive rate) across varying cut-offs. An ROC curve corresponding to greater discriminant capacity of the symptom-based scoring system is located closer to the upper left-hand corner. An ROC curve lying on the diagonal line reflects the performance of a diagnostic test that is no better than chance level. The AUC summarizes the entire location of the ROC curve. The AUC is an effective and combined measure of the sensitivity and specificity that describes the inherent validity of the usefulness of the test in general, where a greater area means a more useful test. If AUC = 1, the symptom-based scoring system is perfect in the differentiation between subjects with and without radiation pneumonitis. This happens when the distribution of the score values for the subjects with and without events do not overlap. In contrast, AUC = 0.5 means that the scoring system is performing no better than chance. Therefore, the AUC can be considered a valuable quantitative measure to prove the diagnostic ability of the symptom-based scoring system. A rough guide for classifying the accuracy of a diagnostic test is the traditional academic point system (AUCs of 0.5–0.6 = fail; 0.6–0.7 poor; 0.7–0.8 = fair, 0.8–0.9 = good and 0.9–1 = excellent). Therefore, any symptom-based score leading to an AUC of at most 0.7 will be rated insufficiently useful and is not considered worth pursuing.

Based on this definition, the following hypothesis system will be subjected to statistical analysis: H0: AUC = 0.7 versus H1: AUC ≠ 0.7.

Non-parametric methods for AUC estimation and testing using the normal approximation of the asymptotic properties of the AUC with standard errors derived by the DeLong, DeLong, and Clarke-Pearson method will be applied [24]. Technically, the SAS version 9.4 (SAS Institute Inc., Cary, NC, USA) LOGISTIC procedure with the ROCCONTRAST statement can be used to estimate the AUC and its 95% confidence limit and to provide the *p*-value for the test mentioned above. A significance level of two-sided 5% is prespecified. If the statistical significance of the AUC is reached, the most informative (optimal) scoring point to predict pneumonitis will be established. Based on discussions with experts, optimality is defined as a score cut-off value of at least 90% and specificity of at least 80%. In addition to this visual selection of a suitable cut-off value, the Youden index (sensitivity + specificity − 1) will be applied to propose an optimal cut-off value for further consideration [25,26].

Due to the limited sample size that could be realistically achieved in this multi-center trial within the given study duration, it was not considered feasible to develop and validate the optimal cut-off value identified within this single study at the same time by dividing the study population into training and validation cohorts. Therefore, the derived cut-off value should be considered a preliminary suggestion, which should be validated in subsequent studies. As a further sensitivity analysis, the relationship between tertiles of the symptom-based scores and the incidence of radiation pneumonitis will be statistically tested using the Jonckheere–Terpstra test, which is a nonparametric test for ordered differences among groups of score values [27,28,29]. It tests the global null hypothesis that the distribution of the response variable does not differ among tertiles. The test is designed to detect alternatives of ordered differences, meaning that the incidence of pneumonitis increases with the tertiles of score values. In addition, the impact of potential risk factors, including radiation dose, co-morbidity (particularly significant cardiovascular disease), history of smoking, radiation doses, and concurrent medication including chemotherapy and immunotherapy on the prevalence of pneumonitis, will be evaluated [4]. The histology of lung cancer (NSCLC or SCLC) was not associated with pneumonitis in previous studies and, therefore, its potential impact will not be separately analyzed [4,6,14].

Based on the participation of highly committed study sites only and our previous trial experience, the rate of missing data can be assumed to be rather low. Furthermore, the primary endpoint consists of the derivation of one patient-specific value from the multiple score values documented during each visit. Based on the scientific question of interest in this single-arm trial, missing values will not be imputed regardless of any intercurrent events (“treatment policy strategy”).

#### 2.6.2. Secondary Objectives

The secondary objective is to determine positive and negative predictive values associated with each scoring point of the symptom-based scoring system. The positive predictive value is the probability that subjects with a high symptom score truly suffer from radiation pneumonitis. The negative predictive value is the probability that subjects with a low symptom score truly don’t suffer from pneumonitis. Thus, the predictive values describe the performance of the scoring system and the relevance for the subjects, whereas sensitivity and specificity describe the intrinsic validity of the test criterion. Since the incidence of subjects experiencing pneumonitis observed in this study reflects the incidence of the target population with the specific inclusion/exclusion criteria (i.e., the number of subjects with pneumonitis is not pre-specified), the positive and negative predictive values for each potential cut-off can be estimated unbiasedly. Point estimates of positive and negative predictive values will be presented.

Patient satisfaction with the symptom-based scoring system is assessed at the end of radiotherapy. Statistical analysis consists of presenting the respective proportions. In case of a dissatisfaction rate >20%, the symptom-based scoring system needs modifications before it can be used in future studies. In case of a dissatisfaction rate >40%, the scoring system will be considered not useful.

### 2.7. Data Management and Monitoring

Data will be collected in accordance with the regulations set out in the Data Protection Act. All the findings from the clinical trial will be stored on electronic data storage devices and treated with the utmost confidentiality. Organizational measures have been taken in order to prevent the data from being communicated to unauthorized persons. Patients will only be identified via their individual patient numbers throughout the entire documentation and evaluation phase, and their full names will not be used.

All data relating to patients will be recorded in a pseudonymous way. Each patient will be identifiable only by the unique patient number, year of birth, and gender. A patient identification list will be kept only in the relevant trial centers and not forwarded to the sponsor. Data collection will be conducted using electronic case report forms. The data will be handled according to the General Data Protection Regulation. The data will be included in one database and analyzed in accordance with a pre-defined statistical analysis plan. Originals of key trial documents, including documentation sheets, will be kept at the trial headquarters (sponsor responsible for the trial) for at least 10 years after the final report. The principal investigator/head of the trial center will keep all administrative documents, the patient identification list, signed informed consent forms, copies of the documentation sheets, and general trial documentation (protocol, amendments) for the abovementioned period. Original patient data must also be kept for the length of time stipulated for the trial centers, but not for less than 10 years.

The Centre for Clinical Trials Lübeck will conduct on-site monitoring at the German sites according to principles of Good Clinical Practice and written standard operating procedures. For initiation, the trial sites will be visited on-site. During the trial, the sites will be visited at regular intervals depending on the recruiting rate and data quality. Informed consent and defined key data will be checked for all patients. The medical file of each patient will be screened for adverse and serious adverse events. Patients’ questionnaires will be checked for their existence. Sites in other countries (than Germany) will be monitored according to the corresponding national regulations. No regular audits are planned, but they may be conducted if necessary. As this trial is not linked to the German pharmaceutical or medicinal product act, inspections by higher federal authorities are not planned. A data monitoring committee is not required since all the patients participating in this trial receive the same treatment for cancer, pneumonitis, and other adverse events as they would receive outside this trial.

Amendments to the study protocol are sent to and require approval from the local ethics committee. Only the coordinating principal investigator may carry out changes. If modifications appear required, the co-investigators should contact the coordinating principal investigator. In case of approved modifications of the study protocol, all the investigators will be informed after the ethics committee approval. Notice has to be confirmed. The coordinating principal investigator will work towards comprehensive internal and external dissemination of project results and knowledge. The coordinating principal investigator, biostatistician, and staff members of the center where the study is performed will create a report regardless of regular or abnormal study termination.

### 2.8. Termination Criteria

#### 2.8.1. Termination Criteria for Individual Patients

The patient may terminate participation in the trial at any time and without giving any reasons. If the patient agrees, follow-up will be performed as planned to receive data, even in case of termination of participation in the study. Otherwise, the reasons for terminating participation in the trial are the same as those for individual termination of treatment. In these cases, follow-up will be completed as planned. The date and reason for termination of treatment must be documented.

#### 2.8.2. Termination of the Trial

The trial will end after the follow-up of 24 weeks after the end of radiotherapy for the last patient. The trial may be stopped prematurely by the principal investigator in case of safety reasons that do not appear to be reasonably justified. In case of premature termination of the trial, the patient and the ethics committee will be informed promptly. Appropriate further therapy and follow-up are assured for the patients.

## 3. Discussion

Radiotherapy of lung cancer may be associated with pneumonitis, a serious adverse event that may even be fatal in approximately 2% of patients [3]. Moreover, in a previous retrospective study, the patients irradiated for lung cancer experiencing severe pneumonitis had worse survival rates than patients without or with mild pneumonitis [30]. Thus, it is important to identify and treat radiation pneumonitis early. A special characteristic of this complication is that it often occurs several weeks or even a few months after completion of the radiotherapy course [4]. Therefore, the symptoms of cough, dyspnea, and fever may be mistaken as signs of acute bronchitis or pneumonia, and patients receive treatment with antibiotics instead of required corticosteroids. To facilitate the diagnosis of radiation pneumonitis and discrimination from other pulmonary diseases, we developed a scoring system in a prospective cohort of 57 patients receiving radiotherapy or chemoradiation for lung cancer [6]. This trial was performed in patients of any age. However, several studies found that elderly patients irradiated for lung cancer have a higher risk of developing pneumonitis when compared to younger patients [7,8,9,10,11,12,13]. Schild et al. presented the results of a secondary analysis of a randomized trial comparing etoposide plus cisplatin and either daily or twice-daily radiotherapy in patients with NSCLC [7]. The retrospective secondary study compared elderly patients aged ≥70 years to younger patients. Grade ≥4 pneumonitis occurred in 6% and 1% of the patients, respectively (*p* = 0.02). In another secondary analysis of a randomized trial of patients with SCLC, grade ≥4 pneumonitis occurred in 6% of patients aged ≥70 years and 1% of patients aged <70 years, respectively (*p* = 0.008) [8]. In an international individual patient data meta-analysis of patients receiving concurrent chemoradiation for NSCLC, the highest risk of pneumonitis was observed in patients aged >65 years receiving carboplatin/paclitaxel [9]. Kharofa et al. investigated patients with NSCLC or SCLC treated with definitive radiotherapy or chemoradiation and found that grade ≥3 pneumonitis occurred significantly more frequently in patients aged ≥70 years than in patients aged <70 (10% vs. 1%, *p* = 0.001) [10]. Moreover, in two retrospective studies from China, older age was identified as a risk factor of pneumonitis in patients irradiated for lung cancer [11,12]. Zhang et al. identified 65 years as the optimal cut-off regarding age and compared patients aged <65 years to patients aged ≥65 years, the inclusion criterion we use for the POLCAR trial [12]. More recently, Socinski et al. presented a secondary analysis of a randomized trial demonstrating that consolidation durvalumab following concurrent chemoradiation for unresectable stage III NSCLC improved progression-free and overall survival [13]. In both the durvalumab group and the placebo group, grade ≥3 pneumonitis occurred more frequently in patients aged ≥70 years than in younger patients.

Despite the prospective design, the study has some limitations. Although the study procedures are clearly defined, the impact of specific pathways of care adopted in the contributing centers cannot be entirely excluded. Patients with significant cognitive deficits unable to appropriately rate their symptoms will not be included in the POLCAR trial, and patients developing neurocognitive decline during the study period will be considered drop-outs. However, neurocognitive decline may not always be detected and, therefore, can affect self-reporting of symptoms. This may also hold true for significant co-morbidity development during the study period. Moreover, seasonal variations in respiratory symptoms may impact the results of the scoring tool.

## 4. Conclusions

Considering the data of previous studies that indicate a significantly higher risk of radiation pneumonitis in elderly patients, a specific scoring tool for this age group appears useful. The POLCAR study aims to establish such a tool that subsequently contributes to the design of a mobile application to be used at home by elderly patients irradiated with lung cancer. The main goal of this study is to identify the optimal scoring point in terms of sensitivity and specificity in order to diagnose radiation pneumonitis and discriminate it from other lung diseases. Moreover, the increase in the scoring points from baseline at the time of pneumonitis is expected to facilitate the diagnosis of pneumonitis. Both the age-specific scoring system and the subsequent mobile application will contribute to an earlier identification of radiation pneumonitis and, therefore, can help avoid severe or fatal forms of this complication. However, the planned mobile application must consider varying levels of technological literacy in the population of elderly cancer patients. The results of this trial will likely not have a significant impact on future radiation treatment planning for elderly patients with lung cancer. The scoring system should be validated in lung cancer patients from outside Europe to evaluate its generalizability. Moreover, future studies may investigate whether the scoring system is also useful for patients with other malignancies receiving thoracic irradiation.

## Figures and Tables

**Table 1:** Inclusion and exclusion criteria.

Inclusion criteria Histologically proven lung cancer Indication for radiotherapy or chemoradiation Mean radiation dose to ipsilateral lung >13 Gy Age ≥65 years Written informed consent Capacity of the patient to contract
Exclusion criteria Expected non-compliance Baseline score of >4 points, as these patients will likely not be able to tolerate the planned treatment, including the full radiation dose

**Table 2:** Schedule of enrolment, interventions, and assessments.

	Screening	Baseline	Weekly During RT	End of RT	Weekly Up to 24 Weeks After RT	End of Study
Informed consent and patient information	X					
Inclusion criteria	X					
Exclusion criteria	X					
Demographics	X					
Medical history and concomitant diseases		X	X	X	X	X
Concomitant medication		X	X	X	X	X
Systemic anticancer treatment		X	X	X	X	X
Physical examination		X				X
Telephone visits					X	
Performance status		X				
Pathology and tumor stage		X				
Upfront surgery		X				
Planned treatment		X				
Lung function test		X	X ^a^	X	X ^a^	X
Chest X-Ray and blood samples (inflammation parameters) ^b^			X ^b^	X ^b^	X ^b^	X ^b^
(Serious) adverse events			X	X	X	X
Treatment given as planned?			X	X	X	X
Symptom-based scoring system (paper version)		X	X	X	X ^c^	X
Satisfaction with the symptom-based score (paper version)				X		

RT = radiotherapy; ^a^ only in case of suspected pneumonitis; ^b^ only if the suspicions of pneumonitis are substantiated after lung function test; ^c^ by phone (telephone visit).

**Table 3:** Scoring points assigned to symptoms potentially associated with pneumonitis.

Symptom	Severity of the Symptom (as Stated by the Patients)	Points
Cough	No	0
	Yes, a little/sometimes	1
	Yes, moderate/regularly	2
	Yes, severe/permanently	3
Shortness of breath	No	0
	Yes, with intense exertion (e.g., climbing stairs)	1
	Yes, with mild exertion (e.g., walking on flat ground)	2
	Yes, at rest	3
Fever	No	0
	Yes, between 37.6 and 38.0 °C	1
	Yes, between 38.1 and 39.0 °C	2
	Yes, higher than 39.0 °C	3

## Data Availability

Further information regarding this trial is available at clinicaltrials.gov (identifier: NCT06480734).

## Abbreviations

The following abbreviations are used in this manuscript:

AUC	Area under the curve
CTCAE	Common terminology criteria for adverse events
DLCO	Diffusing capacity of the lung for carbon monoxide
FEV1	Forced expiratory volume in 1 second
NSCLC	Non-small cell lung cancer
ROC	Receiver operating characteristic
SCLC	Small cell lung cancer
